# Soil-transmitted helminth infections and nutritional status of school-age children, in Mekhoni town, Tigray, Ethiopia

**DOI:** 10.1371/journal.pntd.0013932

**Published:** 2026-02-03

**Authors:** Abel Deres, Brhane Berhe, Tirhas Mulubirhan, Gessessew Bugssa

**Affiliations:** 1 Department of Medical Laboratory Sciences, College of Medicine and Health Sciences, Adigrat University, Adigrat, Ethiopia; 2 Department of Medical Parasitology and Entomology, College of Health Sciences, Mekelle University, Mekelle, Ethiopia; Consejo Nacional de Investigaciones Cientificas y Tecnicas, Fundación Mundo Sano, ARGENTINA

## Abstract

**Background:**

Soil-transmitted helminths pose a significant public health challenge among school-age children in developing countries, such as Ethiopia. This study determined the prevalence of soil-transmitted helminths, associated factors, and nutritional status of school-age children in Mekhoni town, Tigray, Ethiopia, 2025.

**Methods:**

A school-based cross-sectional study design was conducted among 277 schoolchildren in Mekhoni town, Tigray, Ethiopia, from May 2024 to March 2025. A structured questionnaire was used to collect data on demographic characteristics of study participants, and stool samples were collected and processed using direct wet mount and Kato-Katz techniques. Anthropometric measurements were taken, and anthropometric indices were generated using the WHO AnthroPlus software to determine the nutritional status of schoolchildren. The data were analyzed using SPSS 27 software. Descriptive statistics were applied to present the data using frequency, tables, and figures. Bivariate logistic regressions were used to examine the correlation between the dependent variable and the individual independent variable, and multivariate logistic regressions were performed to determine the independent effect of the main explanatory variable on the outcomes of interest. A P-value < 0.05 was considered statistically significant.

**Results:**

The overall prevalence of soil-transmitted helminth infections was 16.2% (n = 45), with *Ascaris lumbricoides* being the most identified parasite, at 10.1% (n = 28), followed by *Trichuris trichiura* at 6.1% (n = 17), and hookworms, at 2.9% (n = 8). Of the infected children, 31 (18.7%) were males, and 14 (12.6%) were females. The number of infections is also higher among children within the age group of 5–10, 22 (18.4%), than among those 11–14 years old, 23 (14.65%). Factors significantly associated with increased odds of infection were having large family size (AOR = 2.56, 95% CI: 1.18-5.55, P = 0.017), unclean fingernails (AOR = 2.63, 95% CI: 1.14-6.03, P = 0.022), untrimmed fingernails (AOR = 2.25, 95% CI: 1.006-5.03, P = 0.002), and lack of hand washing after visiting a toilet (AOR = 0.29, 95% CI: 0.13-0.63, P = 0.002). The overall prevalence of undernutrition was 37.5% (n = 104), with 22.2% being underweight (n = 117), 27.8% stunted (n = 77), and 11.19% wasted (n = 31). There was no statistically significant association between STH infection and nutritional status among study subjects.

**Conclusion:**

The prevalence of STH infections was less than 20% (n = 45). The most common species of STH infections identified were *Ascaris lumbricoides*. The study also revealed a high prevalence of undernutrition among school-age children. Ensuring access to clean toilets and hand-washing facilities in the schools, introducing a school health and nutrition program are vital.

## Author summary

Soil-transmitted helminth (STH) infections are a significant public health concern, especially in developing countries. This study examines the prevalence of STH infections and the nutritional status of school-age children in Mekhoni Town, Tigray, Ethiopia. The research found that the overall prevalence of STH infection in the study population was 16.2% (n = 45). Factors such as large family size, uncleaned fingernails, untrimmed fingernails, and handwashing practice after visiting a toilet were identified as significant contributors to the likelihood of STH infection. On the other hand, the study revealed that 37.5% (n = 104) of the children were undernourished, yet no statistical association was found between STH infection and undernutrition. These findings highlight the importance of improving hygiene practices and further exploring the relationship between STH infection and nutritional status in this region.

## Introduction

*Ascaris lumbricoides*, *Trichuris trichiura*, and hookworms are the three main parasitic worms that cause soil-transmitted helminthic infections (STHs) [1]. Although not routinely included in global or national deworming programs, *Strongyloides stercoralis*, which is transmitted through skin penetration by infective larvae, remains an important STH species and has been reported to be highly prevalent in Ethiopia [2]. *A. lumbricoides* and *T. trichiura* are transmitted through the fecal-oral route, where individuals become infected by ingesting eggs from contaminated water, surfaces, hands, or food. Hookworm infections are spread through the skin by contact with soil contaminated with infective larvae [3], ingestion of larvae [4], or through the transmammary route [5]. Low income, inadequate personal hygiene, poor environmental sanitation, restricted availability of clean water, and tropical climate are the factors linked with infection of STH [6]. South Asia, Southeast Asia, and Sub-Saharan Africa are the regions with the highest burden [4]. These parasitic infections continue to pose significant public health challenges, particularly among school-age children (SAC) in developing nations with limited resources [7,8]. Approximately 2 billion individuals worldwide carry at least one type of STH infection (with *A. lumbricoides*, 1 billion, *T. trichiura,* 800 million, and hookworm, 740 million). Besides, around 4 billion people are susceptible to contracting STH infections globally [9,10], causing an approximate 4.98 million years of disability adjusted life years (DALYs). Consequently, around 300 million individuals experience severe morbidity due to STH, leading to annual deaths ranging from 10,000 to 135,000 [11]. In Africa, it is estimated that around 90 million SAC are believed to have STH infections [12]. In Ethiopia, impacting over 79 million people, with approximately 25 million being SAC [13]. The nationwide occurrence rates of hookworms, *A. lumbricoides*, and *T. trichiura* stood at 16%, 37%, and 30%, respectively [8].

Despite the introduction of deworming initiatives and improvements in water, sanitation, and hygiene (WASH) practices in the region, the prevalence of STH infections among school-age children in Tigray remains approximately 10% [14]. The continued existence of these infections indicates that the root causes, such as poor sanitation, lack of access to clean water, and limited health education, have not been sufficiently addressed. Furthermore, in regions like Tigray, where there are internally displaced people due to war, managing infectious diseases becomes exceedingly complex and challenging [15]. On the other hand, the prevalence of stunting, underweight, and wasting among primary school-age children in Ethiopia was 21.3%, 18.2%, and 17.7% respectively [16]. Malnutrition and STH frequently occur together in the same area, affecting the same individuals simultaneously and perpetuating each other [17].

According to WHO recommendations, preventive chemotherapy (PC) through Mass Drug Administration (MDA) is the main strategy for controlling STH in school-aged children. WHO recommends annual deworming in areas where STH prevalence is between 20% and 50%, and biannual deworming where prevalence exceeds 50%. In settings with a prevalence below 20, routine MDA is not required, and selective treatment is advised [18].

In Ethiopia, school-based deworming for STH has been implemented since 2015 as part of the national NTD control program, and significant progress has been achieved in expanding MDA coverage among school-aged children. However, despite these national efforts, some segments of the population remain unreached [19]. In the present study area, specific data on MDA implementation is not available, highlighting the need for local epidemiological evidence.

The recent destructive war in the Tigray region of Ethiopia has probably worsened the challenges associated with STH infections and malnutrition among SAC in the study area. The interruption of essential services, displacement of communities, and breakdown of health care and sanitation infrastructure in the Tigray region could have heightened the prevalence and seriousness of these public health concerns. Undertaking this research in the post-conflict context to determine the prevalence of soil-transmitted helminths, associated factors, and nutritional status among school-age children in Mekhoni town, Tigray, Northern Ethiopia will produce vital evidence to steer the implementation of the recovery and rehabilitation endeavors, guaranteeing that the health and nutritional requirements of the vulnerable school-age population are adequately met.

## Methods

### Ethical statement

Ethical clearance was obtained from Mekelle University, College of Health Sciences Ethical Review Committee. Furthermore, a permission letter was obtained from the Tigray Regional Health Bureau and the Mekhoni Wereda Health office. Besides, further permission was obtained from the selected school’s administrator. Moreover, written informed consent was obtained from the families and/ or guardians of all participating children, and verbal assent was obtained from the children. Each piece of data was kept confidential. All children who were found positive for STH had their results communicated to their families and were linked to the nearest health facility for treatment according to national deworming guidelines.

### Study setting

**Fig 1:**
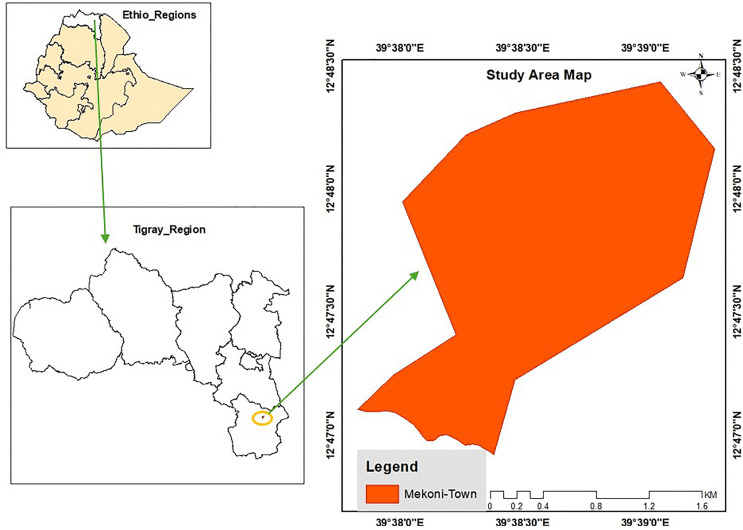
Map of Mekhoni town (Map was created using ArcGIS 10.8; shapefile source is Diva-GIS, available at https://diva-gis.org/).

Mekhoni is a town situated 657 kilometers north of Addis Ababa, the capital city of Ethiopia, and 126 kilometers south of Mekelle, the capital city of the Tigray National Regional State. It is positioned at coordinates 12^0^47’30’‘N and 12^0^48’30’‘N latitude, and 39^0^38’00’‘ and 39^0^39’00’‘E longitude (Fig 1). Mekhoni sits at the center of Raya Azebo Woreda, which is bordered to the south by Alamata Woreda, to the north by Hintalo-Wajirat Woreda, to the west by Enda mekoni and Emba-Alaje, and to the east by the Afar Regional State of Ethiopia.

The district has a bimodal rainfall pattern, with light rains from February to April and heavier rains between July and September. On average, annual rainfall is about 724 mm. In the western highlands, daily temperature typically ranges from 13.93 °C to 18.3 °C, while in the valley, it is warmer, ranging from 19.64 °C to 23.44 °C. Around 90% of the district is considered “midland”, lying between 1,500 and 2,300 m above sea level, while the remaining 10% falls into the “lowland” category, below 1,500 m [20].

According to the projected population data from the Central Statistical Agency (CSA), Raya Azebo Wereda has a total population of 176,401, comprising 86,810 males and 88,591 females. Of these, 39,101 live in urban areas, while the majority, 137,300, reside in rural areas [21].

The economy of the population is heavily dependent on agricultural activities, which serve as the primary source of livelihood for most of its population. The region’s fertile lands support the cultivation of crops such as Sorghum, teff, and maize, while livestock farming also plays an important role in the local economy [22]. The town has a primary hospital, which provides inpatient and outpatient medical services to residents of Mekhoni town and Raya Azebo wereda. The town has four elementary schools, all of which have suffered significant damage to their sanitation and water facilities because of the recent devastating war.

### Study design, objective, and study period

A school-based cross-sectional study was conducted to determine the prevalence of soil-transmitted helminths, associated factors, and nutritional status among school-age children in Mekhoni town, Tigray, Northern Ethiopia, from May 2024 to March 2025.

### Eligibility criteria

School-age children who were willing to participate, provided stool samples, and obtained consent from their parents or legal guardians were included in the study.

### Sample size and sampling technique

A sample size was determined using the single population proportion statistical formula, as shown below.


$$n=Z\alpha /{\text{2}}^{\text{2}}\frac{P(\text{1}-P)}{d^{\text{2}}}$$

where n = required sample size, Z is the statistic that corresponds to the desired confidence level (95%), P is the estimated population proportion, and d is the margin of error (the desired precision of the estimate). Taking a prevalence of 21% as the best available estimate for the prevalence of STH infections from a previous study [23], a margin of error of 5%, a z-score of 1.96, and adding 10% for non-response resulted in a final sample size of 285.

Two elementary schools were chosen from the four available in Mekhoni town using simple random sampling. Once the schools were selected, students were stratified by their educational levels (grades 1–8). The allocation of schoolchildren to schools and grade levels was done proportionally based on the number of students in each school and grade [24]. Sections within the schools were selected using a lottery method. A roster containing the list of students was compiled as the sampling frame. To select study participants for each grade level, systematic random sampling was employed, following proportional allocation. The sampling interval (K) was calculated by dividing the total number of units in the population (N) by the desired sample size (n), resulting in a sampling interval of 11 (K = 11) across the two schools.

**Fig 2:**
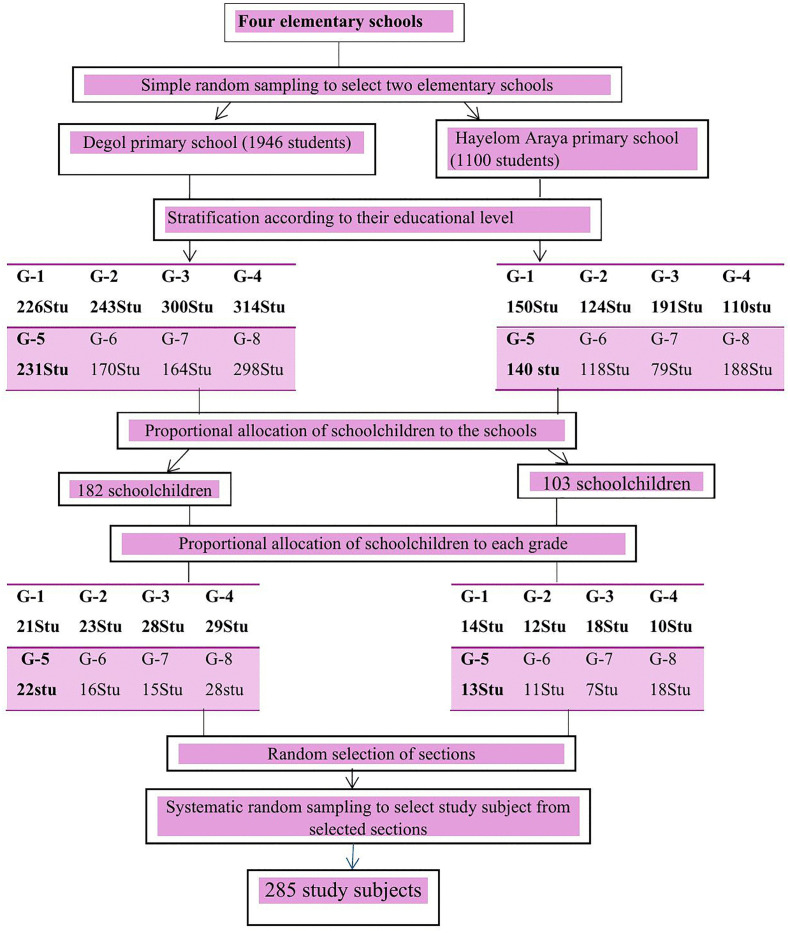
Schematic representation of sampling procedure.

The first student was randomly selected from the Kth interval, and subsequent participants were chosen at every Kth interval thereafter (Fig 2).

### Data collection procedure

#### Questionnaire.

Data on demographic characteristics, hygiene, and sanitation of study participants were collected using a structured and pre-tested questionnaire. Household information was obtained from parents or guardians, while child hygiene practice was asked directly to the child. All interviews were conducted at the school. The questionnaire was developed in English based on previous research works and translated into the local language (Tigrigna). The face-to-face interview was conducted by trained data collectors.

#### Anthropometric measurement.

Using standardized anthropometric measurements, weight and height were taken for all study participants. For weight measurement, children were asked to remove their shoes, wear light clothes, and remove accessories. Then, trained data collectors measured children’s weight on a calibrated portable digital scale (FAZZINI S759) and recorded the value to the nearest 0.1 kilograms. For height measurement, children were instructed to stand upright with their shoulders aligned, arms at their sides, thighs and heels touching comfortably together, the buttocks, scapulae, and head were positioned against the vertical backboard with a sliding head bar. The height was then recorded to the nearest 0.1 centimeter [25].

#### Parasitological investigation.

Labeled stool containers with tight covers bearing serial numbers of the subjects were supplied for the study participants. Fecal specimens were processed by wet mount and Kato-Katz techniques. Fresh stool samples (approximately 2 mg of stool) were put on a slide with the wooden applicator and emulsified with a drop of physiological saline (0.85%). The preparation was then covered with a cover slide and examined at 10× and 40 × microscopic objectives [26]. Thick smear was prepared using the Kato-Katz template, which delivers 41.7 mg of stool, and the smear was transported to Mekhoni Primary Hospital for further analysis. The slides were examined by an experienced laboratory technologist under 10-x and 40-x objective lenses for the presence of eggs of STH. Hookworm ova detection was performed by examination of the Kato-Katz slide within one hour of stool collection and its preparation. Furthermore, all eggs of STHs were counted under a microscope and reported as the number of eggs per gram of stool(EPG) by multiplying by the appropriate factor of 24 [27]. The intensity of the infection was estimated based on the cut-off value for the classification of infection intensity [28]. As light, moderate, and heavy infections. 1–4999 epg, 5000–49999 epg, and >50000 epg, respectively for *A. lumbericoides* for *T. trichuira*, 1–999 epg, 1000–9999 epg, and >10000 epg, and for hookworm, 1–1999 epg, 2000–3999 epg, and >4000 epg according to WHO cut-off values [29].

### Data quality assurance and management

The Questionnaire was pre-tested in Baro Elementary School before actual data collection. The data collected were checked for consistency and accuracy daily. All the laboratory procedures were conducted as per the Standard Operating Procedures. Two slides were prepared from each sample and were independently examined by two Medical Laboratory Technologists. Additionally, 10% of the samples were randomly chosen and re-checked by senior medical Laboratory Technologists.

### Data analysis and interpretation

All data was recorded by hard copy. After checking for completeness, data were coded and entered, using SPSS (Statistical Package for Social Sciences statistical software) version 27. Then Descriptive statistics were applied to present the data using frequency, tables, and figures. Binary logistic regression was employed to show the correlation of the dependent variable with the individual independent variable. Potential confounders were identified based on previous literature [23]. Variables with a p-value <0.25 in the bivariable analysis were included in the multivariable logistic regression model. Multivariate analysis was done to identify the independent effect of the main explanatory variable on the outcomes of interest after adjusting for several other confounding variables. A P-value of <0.05 was considered indicative of a statistically significant difference. The results of the logistic regression indicated that the chosen model was a good fit, as evidenced by the Hosmer-Lemeshow goodness-of-fit test, which yielded a P-value of 0.08. WHO AnthroPlus 2009 (v 1.0.4) was used to calculate weight-for-age z-scores (WAZ), height-for-age z-scores (HAZ), and BMI-for-age z-scores (BAZ). Children < -2SD were classified as Under-weight (WAZ < -2SD), Stunted (HAZ < -2SD), and Wasted (BAZ < -2SD) [30].

### Operational definitions

**School-age children:** children who attend primary school (one to eighth grade) and are in the age group of 5–14 years.
**Anthropometric measurements:** are systematic measurements of the variation of physical dimensions and composition of the human body.
**Nutritional status:** is the child’s state of the body, which is determined by anthropometric measurement indices as normal, stunted, wasted, or underweight based on the WHO standard reference 2007
**Undernutrition:** is a poor nutritional status of primary schoolchildren, which is expressed in anthropometric indices when the Z-scores for the WHO standards of 2007 are less than minus two (Z-scores < -2SD)
**Underweight**: weight-for-age z-scores below -2SD
**Severe underweight:** weight-for-age z-scores below -3SD
**Stunting**: height-for-age z-scores below -2SD
**Severe stunted:** height-for-age z-score below -3SD
**Wasting**: BMI-for-age z-scores below -2SD
**Severe wasted**: BMI-for-age z-scores below -3SD

## Results

### Socio-demographic, hygienic, and environmental characteristics of the study participants

A total of 277 study participants were enrolled, with a response rate of 97.2%. Representing both Degol and Hayelom Araya Elementary Schools. Most were in the older age group, and the sex distribution was slightly male-dominant. In addition, information was collected on the key hygiene behaviors and household environmental conditions, including handwashing practices, access to safe water, waste disposal methods, and toilet availability. These characteristics are summarized in the combined table below (Table 1).

**Table 1:** Socio-demographic, hygienic, and environmental characteristics of school-age children, Mekhoni town, Tigray, Ethiopia, May 2024 to March 2025 (N = 277).

Variables	Category	Frequency (%)
**School**	Degol	176 (63.5)
	Hayelom Araya	101 (36.5)
**Sex**	Male	166 (59.9)
	Female	111 (40.1)
**Age in years**	5-10	120 (43.3)
	11-14	157 (56.7)
**Religion**	Orthodox	234 (84.5)
	Muslim	43 (15.5)
**Grade**	1-4	150 (54.2)
	5-8	127 (45.8)
**Monthly family income (ETB)**	<1000	39 (14.1)
	1000-2000	25 (9)
	>2000	213 (76.9)
**Family size**	≤5	137 (49.5)
	>5	140 (50.5)
**Mother’s educational status**	No formal education	97 (35)
	1-8	97 (35)
	≥9	83 (30)
**Mother’s occupation**	Merchant	124 (44.8)
	Civil servant	65 (23.5)
	Housewife	61 (22)
	Daily labourer	27 (9.7)
**Father’s educational status**	No formal education	103 (37.2)
	1-8	90 (32.5)
	≥9	84 (30.3)
**Fathers’ occupation**	Merchant	102 (36.8)
	Farmer	58 (20.9)
	Civil servant	68 (24.5)
	Daily labourer	49 (17.7)
**Wash hands before meal**	Yes	259 (93.5)
	No	18 (6.5)
**Frequency of washing hand before meal**	Always	228 (82.3)
	Sometimes	31 (11.2)
**Wash hands after toilet**	Yes	271 (97.8)
	No	6 (2.2)
**Frequency of washing hands after toilet**	Always	217 (78.3)
	Sometimes	54 (19.5)
**Using water and/or soap after toilet**	Using water only	92 (33.2)
	Using water and soap	179 (64.6)
**Fingernail cleanness**	Clean	202 (72.9)
	Not clean	75 (27.1)
**Trimming fingernails**	Trimmed	175 (63.2)
	Not trimmed	102 (36.8)
**Shoe wearing habit**	Always	275 (99.3)
	Sometimes	2 (0.7)
**Habit of eating unwashed/ undercooked vegetables**	Yes	31 (11.2)
	No	246 (88.8)
**Availability of toilet**	Yes	256 (92.4)
	No	21 (7.6)
**Type of toilet**	Traditional pit latrine	138 (49.8)
	Ventilated improved	118 (42.6)
**Solid waste disposal habit**	Burry underground	17 (6.1)
	Open field	19 (6.9)
	Incinerate	17 (6.1)
	By municipality	224 (80.9)
**Water source for drinking**	Well water	77 (27.8)
	Tap water	200 (72.2)
**Water source for bathing**	Well water	77 (27.8)
	Tap water	200 (72.2)
**Water source for washing cloth**	Well water	77 (27.8)
	Tap water	200 (72.2)

### Prevalence of soil-transmitted helminths

A total of 45 (16.2%) study participants were found to be positive for one or more STH infections. Moreover, *A. lumbricoides* represented the most dominant STH parasite, affecting 10.1% (n = 28) of the study participants. *T. trichiura* and hookworm were detected in 6.1% (n = 17) and 2.9% (n = 8) of the schoolchildren, respectively. Of the infected children, 31 (18.7%) were males, and 14 (12.6%) were females (Table 2).

**Table 2:** Frequency of soil-transmitted helminths identified among schoolchildren in Mekhoni town, Tigray, Ethiopia, May 2024 to March 2025 (N = 277).

STH species	Overall prevalence (%)	Single infection	Co-infection
***A. lumbricoides***	28 (10.1)	20	6 (with Trichuris), 2 (with Hookworm)
***T. trichiura***	17 (6.1)	11	6 (with Ascaris)
***Hookworm***	8 (2.9)	6	2 (with Ascaris)
**Total infected children**	45 (16.2)	37	8

### STH co-infections and intensity

The prevalence of single STH infection was 82.2% (37/45). A single infection by *A. lumbricoides* was the most predominant parasite, constituting 54% (20/37) of the infections. Co-infection by *A. lumbricoides* and *T. trichiura* was 75% (6/8) whereas *A. lumbricoides* and hookworm co-infections were 25% (2/8). The proportions of light infections for *A. lumbricoides* and Hookworm were 100%; only 2 cases of *T. trichuira* were with moderate infection intensity (Table 3).

**Table 3:** Intensity of infection in schoolchildren with STH in Mekhoni town, Tigray, Ethiopia, May 2024 to March 2025 (N = 277).

Type of STH infection	Arithmetic mean EPG (SD)	Geometric mean EPG	Class of infection intensity			
			Light (%)	Moderate (%)	Heavy (%)	Total (%)
**Ascariasis**	1122 (±394)	1049.7	28 (100)	0	0	28 (100)
**Trichiurasis**	598 (± 273)	534	15 (88.2%)	2 (11.8)	0	17 (100)
**Hookworms**	561 (± 179.6	534.4	8 (100)	0	0	8 (100)

Epg = eggs per gram of stool.

### Nutritional status and STH infection

The overall prevalence of under-nutrition was 37.5% (N = 104). Besides, 26 (22.2%) children aged 5–10 years were underweight, 77 (27.8%) of the children were stunted, and 31 (11.19%) of the children were wasted. The prevalence of severe forms of underweight among children aged 5–10 years (WAZ < -3SD), severe stunting (HAZ < -3SD), and wasting (BAZ < -3SD) was 5 (4.3%), 19 (6.9%) and 9 (3.2%) respectively.

Of the children with undernutrition, 26 (25%) of them were with STH infections. Among the children infected with STH, 27.3% were underweight (n = 117), 44.4% were stunted, and 20% were wasted. In comparison, the prevalence of underweight, stunting, and wasting among healthy children was 20% (n = 117), 24.5%, and 9.5%, respectively (Table 4).

**Table 4:** Nutritional status of Schoolchildren in Mekhoni town, Tigray, Ethiopia, May 2024 to March 2025 (N = 277).

Variable	Age group	STH status	N (%)	Total (%)	Median (IQR)
**Undernutrition**	5-10	Positive	8 (2.9)	104 (37.5)	
		Negative	34 (12.3)		
	11-14	Positive	18 (6.5)		
		Negative	44 (15.9)		
WAZ (n = 117*)					-1.44 (-1.91-(-.55)
**Below -2SD underweight**	5-10	Positive	6 (5.1)	26 (22.2)	
		Negative	20 (17.1)		
	11-14	Positive	NA		
		Negative	NA		
**Below -3SD severe underweight**	5-10	Positive	0 (0)	5 (4.3)	
		Negative	5 (4.3)		
	11-14	Positive	NA		
		Negative	NA		
**HAZ**					-1.38 (-2.08-(-.76)
**Below -2SD stunted**	5-10	Positive	6 (2.2)	77 (27.8)	
		Negative	22 (7.9)		
	11-14	Positive	14 (5.1)		
		Negative	35 (12.6)		
**Below -3SD (severe stunted)**	5-10	Positive	1 (0.4)	19 (6.9)	
		Negative	5 (1.8)		
	11-14	Positive	3 (1.1)		
		Negative	10 (3.6)		
**BAZ**					-0.69 (-1.4-(-.09)
**Below -2SD wasted**	5-10	Positive	0 (0)	31 (11.9)	
		Negative	12 (4.3)		
	11-14	Positive	9 (3.2)		
		Negative	10 (3.6)		
**Below -3SD (severe wasted)**	5-10	positive	0 (0)	9 (3.2)	
		Negative	3 (1.1)		
	11-14	Positive	3 (1.1)		
		Negative	3 (1.1)		

Height-for-age Z-score (HAZ), Weight-for-age Z-score (WAZ), Body-mass-index-for-age Z-score (BAZ), Not applicable (NA).

*For WAZ (an indicator of underweight), only calculated for individual ≤ 10 years of age (age group 10 years covers up to age 120 complete months), n = 117.

### Factors associated with STH infection

The family size of study participants, hand washing method after toilet, cleanness of fingernails, and trimming of fingernails were identified as independent factors associated with STH infection at a significant level of 0.05. Family size emerged as a strong predictor of STH, with participants from households of more than five members being more likely to be infected by STH than participants having a family size ≤5 (AOR = 2.56, 95% CI: 1.18-5.55, P = 0.017). Participants who washed their hands with both water and soap after using the toilet were 71% less likely to be infected with STH compared to those who washed with water only (AOR = 0.29, 95% CI: 0.13-0.63, P = 0.002). Additionally, children who did not clean their fingernails were 2.63 times more likely to be infected than those who did (AOR = 2.63, 95% CI: 1.14-6.03, P = 0.022). Likewise, children with untrimmed fingernails had a higher likelihood of STH infection compared to those with trimmed nails (AOR = 2.25, 95% CI: 1.006-5.03, P = 0.002). In bivariate logistic regression, undernutrition, stunting, and wasting were found to be statistically significant when STH infection status was considered as the outcome variable. However, these factors were no longer significant after adjusting for confounders in multivariate logistic regression (Table 5).

**Table 5:** Risk factors for soil-transmitted helminthic infections among schoolchildren, Mekhoni, Tigray, Ethiopia, May 2024 to March 2025 (N = 277).

Variable	Category	STH infection		COR (95% CI)	P	AOR (95% CI)	P
		Presence (%)	Absence (%)				
**Sex**	Male	31 (18.7)	135 (81.3)	1			
	Female	14 (12.6)	97 (87.4)	0.6 (0.3-1.24)	0.183	1.06 (0.47-2.37	0.88
**Age group**	5-10	22 (18.33)	98 (81.67)	1			
	11-14	23 (14.65)	134 (85.35)	0.765 (0.4-1.45)	0.411		
**Father’s occupation**	Merchant	12 (11.8)	90 (88.2)	1			
	Farmer	10 (17.2)	48 (82.7)	1.56 (0.62-3.87)	0.336	1.76 (0.63-4.91)	0.279
	Civil servant	13 (19.1)	55 (80.9)	1.77 (0.75-4.16)	0.189	2.1 (0.8-5.9)	0.12
	Daily labourer	10 (20.4)	39 (79.6)	1.92 (0.76-4.82)	0.163	1.46 (0.49-4.29)	0.49
**Family size**	≤5	14 (10.2)	123 (89.8)	1			
	>5	31 (22.1)	109 (87.9)	2.49 (1.26-4.94)	0.008	2.56 (1.18-5.55)	0.017*
**Using water and/or soap after toilet**	Using water only	27 (28.7)	65 (71.3)	1			
	Using water and soap	17 (9.5)	162 (90.5)	0.25 (0.12-0.49)	0.001	0.29 (0.13-0.63)	0.002*
**Fingernail cleanness**	Yes	21 (10.4)	181 (89.6)	1			
	No	24 (32)	51 (68)	4.05 (2.09-7.87)	0.001	2.63 (1.14-6.03)	0.022*
**Fingernail trimmed**	Yes	20 (11.4)	155 (88.6)	1			
	No	25 (24.5)	77 (75.5)	2.51 (1.31-4.81)	0.005	2.25 (1.006-5.03)	0.048*
**Nutritional status**	Normal	19 (11)	154 (89)	1			
	Under nutrition	26 (25)	78 (75)	2.7 (1.40-5.18)	0.003	1.45 (0.27-7.6)	0.65
**Stunting**	No	25 (12.5)	175 (87.5)	1			
	Yes	20 (26)	57 (74)	2.45 (1.27-4.75)	0.008	1.41 (0.30-6.67)	0.65
**Wasting**	No	36 (14.6)	210 (85.4)	1			
	Yes	9 (29)	22 (71)	2.38 (1.01-5.59)	0.045	1.91 (0.45-8.07)	0.37

## Discussion

A prevalence of 16.2% in our study area shows that transmission has been reduced compared with historically high levels in Ethiopia [13,31], but it has not been eliminated. By WHO programmatic thresholds, this sits below the 20% cut-off commonly used to trigger district-wide annual MDA [18], yet it remains high enough to warrant caution. Evidence from national mapping and recent reviews shows that Ethiopia’s progress has been uneven; many woredas have improved after repeated school-based preventive chemotherapy, while others continue to harbor persistent pockets of STH, often linked to uneven coverage and poor WASH conditions [31,32].

Within this context of uneven national progress, the prevalence in our study area is higher than reported rates from Wera-baye (6.6%) [33], Enderta wereda (6.5%) [34], Gurage zone (9.5%) [35], and Cameroon (2.2%) [36]. However, the prevalence in this study is lower than that observed in Fogera district (30.30%) [37], Yirgachefe (54.5%) [38], Dara Mallo and Uba Debretsehay districts (33.2%) [39], Mettu (84.4%) [40], and Jimma (55%) [30]. On the other hand, it is similar to findings from studies conducted in Adola (16.1%) [24], Ambo (12.8%) [41], and Western Kenya (17.0%) [42]. Variation in socio-demographic and economic factors, as well as differences in awareness regarding STH exposure, transmission, and prevention, may explain the discrepancies in prevalence between this study area and the others.

In terms of species, *A. lumbericoides* was the most common STH infection, with a prevalence rate of 10.1%. This is higher than the findings in Medebay Zana (1.71%) [43] and Gurage zone (3%) [35]. But lower than a study from Yirgachefe (21.7%) [38] and Mettu (39%) [40]. Though the prevalence of *A. lumbericoides* in the current study is consistent with previous studies in Dembecha (11.4%) [23], and Hawassa (11.2%) [44]. *T. trichuira* and Hookworm had prevalence rates of 6.1% and 2.9% respectively, which is higher than the one observed in Wera-abaye (1.6%, 0%) [33], and Enderta wereda (0.21%, 0.42%) [34]. Moreover, the prevalence rate in this study aligns with research conducted in Hawassa (4.5%, 2.7%) [44]. In contrast, hookworm infection is much lower than the study conducted in Durebte town (46.9%) [9]. The variation in the prevalence of specific parasites may be linked to variations in the suitability of the macro and microenvironments for the parasites across different regions, study time frame, geographical locations, urbanization, and lack of public health education, deworming practices, as well as economic, social, and cultural factors. As it has been described in literature [45].

This study found that factors such as family size, hand washing practices after using the toilet, fingernail cleanness, and trimmed fingernails were significantly linked to STH infections. Similarly, studies in Dembecha [23], Hawassa [44], and Ambo [41] also identified family size as a key risk factor for STH infection. In this study, children who washed their hands with both water and soap after using the toilet were 71% less likely to be infected with STH infections (AOR = 0.29, 95 CI (0.13-0.63), P = 0.002). Similar to a study conducted in Lurambi, Kenya [46]. Another report also highlighted the importance of nail trimming and cleanliness in relation to STH infection, which aligns with our findings [8].

Among the schoolchildren investigated, 37.5% were found to be suffering from undernutrition, which is higher than the rates in Addis Ababa (30.9%) [47] and Yirgachefe (28.9%) [38]. But lower than the rate reported from Debremarkos (56.2%) [48]. The stunting prevalence was found to be 27.8%, which is consistent with findings from studies in Agulae (25.5%) [49] and Jimma (21%) [30]. However, it was higher than the prevalence reported in Chencha (8.9%) [17]. In contrast, the prevalence was lower than studies conducted in Hawzen (33.3%) [50], Arbaminch (41.9%) [25], and Mulo district (42.4%) [51]. The prevalence of wasting was 11.9%. Which is lower compared to the study conducted in Agulae (44%) [49]. In spite of that, our result is higher than the rates reported in Yirgachefe (5.2%) [38] and Jimma (6.9%) [30]. The prevalence of underweight in the population being studied was found to be 22.2%, which is lower than the results reported in Agulae (55%) [49] and Hawzen (32.2%) [50]. On the other hand, it is higher than the prevalence observed in Addis Ababa (15.4%) [47], Yirgachefe (12.9%) [38], and Mettu (5.1%) [40]. The disparity in stunting, wasting, and underweight prevalence across different regions is possibly due to differences in factors such as socio-economic status, living conditions, improper child feeding practices, geographic differences, variations in agricultural productivity, urbanization, and healthcare for the children.

The lack of association between nutritional status and STH infection in this study is consistent with findings from Yirgachefe [38] and Chencha [17], unlike the study conducted in Jimma [30] and Mettu [40], in which *T. trichiura* is a predictor of stunting and undernutrition, respectively.

## Limitations

This study shares the limitations of a cross-sectional study design, which does not establish a cause-and-effect relationship between dependent and independent variables. Additionally, the Kato-Katz technique has limited sensitivity, particularly for light-intensity infections, which may have led to underestimation of the true prevalence of STH infections. *Strongyloides sterocolralis* was not assessed, as it requires different diagnostic methods and a different treatment regimen than the STH species targeted by WHO’s preventive chemotherapy programs. Furthermore, the study relied solely on anthropometric measurements to assess nutritional status, without biochemical and dietary assessment.

## Conclusion

The current study found that the prevalence of STH infections is less than 20%, indicating a low-risk category based on WHO classifications. The most common species of STH infections identified were *Ascaris lumbricoides*. Additionally, STH infections were linked to factors such as large family size, nail cleanness, nail trimming, and hand-washing practices after using the toilet. The study also revealed a high prevalence of undernutrition among school-age children; however, there was no significant difference in the nutritional status of children with or without STH infections. Ensuring access to clean toilets and hand-washing facilities in the school, promoting hygiene awareness and teaching good practice, and introducing a school health and nutrition program are vital to enhance the health and nutritional status of the schoolchildren.

## Supporting information

S1 File Questionnaire to assess socio-demographic, hygienic, and environmental factors associated with STH infection. (DOCX)

S1 Table All socio-demographic, hygiene, and environmental factors, along with statistical values. (DOCX)

S1 Data Raw SPSS dataset. (SAV)

## References

[1] Salma Z, Renald RBY, Husada D, Basuki S. Soil-transmitted helminthes infection and nutritional status of elementary school children in Sorong District, West Papua, Indonesia. Indones J Trop Infect Dis. 9(2).

[2] Jember TH, Amor A, Nibret E, Munshea A, Flores-Chavez M, Ta-Tang T-H, et al. Prevalence of Strongyloides stercoralis infection and associated clinical symptoms among schoolchildren living in different altitudes of Amhara National Regional State, northwest Ethiopia. PLoS Negl Trop Dis. 2022;16(4):e0010299. doi: 10.1371/journal.pntd.0010299 35482629 PMC9049318

[3] Maddren R, Phillips A, Ower A, Landeryou T, Mengistu B, Anjulo U, et al. Soil-transmitted helminths and schistosome infections in Ethiopia: a systematic review of progress in their control over the past 20 years. Parasit Vectors. 2021;14(1):97. doi: 10.1186/s13071-021-04680-0 33546757 PMC7866680

[4] Steinbaum L, Kwong LH, Ercumen A, Negash MS, Lovely AJ, Njenga SM, et al. Detecting and enumerating soil-transmitted helminth eggs in soil: New method development and results from field testing in Kenya and Bangladesh. PLoS Negl Trop Dis. 2017;11(4):e0005522. doi: 10.1371/journal.pntd.0005522 28379956 PMC5393894

[5] Caldrer S, Ursini T, Santucci B, Motta L, Angheben A. Soil-Transmitted Helminths and Anaemia: A Neglected Association Outside the Tropics. Microorganisms. 2022;10(5):1027. doi: 10.3390/microorganisms10051027 35630469 PMC9143297

[6] Wiryadana KA, Putra IWAS, Rahayu PDS, Pradnyana MM, Adelaida ML, Sudarmaja IM. Risk factors of soil-transmitted helminth infection among elementary school students. Paediatr Indones. 2018;57(6):295.

[7] Imam A, Farouk ZL, Hassan-Hanga F, Ihesiulor UG. A comparative cross-sectional study of prevalence and intensity of soil-transmitted helminthic infection between healthy and severe acutely malnourished pre-school aged children in Kano, Northern Nigeria. BMC Infect Dis. 2019;19(1):121. doi: 10.1186/s12879-019-3755-3 30727974 PMC6364394

[8] Aemiro A, Menkir S, Tegen D, Tola G. Prevalence of Soil-Transmitted Helminthes and Associated Risk Factors Among People of Ethiopia: A Systematic Review and Meta-Analysis. Infect Dis (Auckl). 2022;15:11786337211055437. doi: 10.1177/11786337211055437 35356097 PMC8958720

[9] Alelign T, Degarege A, Erko B. Soil-Transmitted Helminth Infections and Associated Risk Factors among Schoolchildren in Durbete Town, Northwestern Ethiopia. J Parasitol Res. 2015;2015:641602. doi: 10.1155/2015/641602 26161265 PMC4487929

[10] Anuar TS, Salleh FM, Moktar N. Soil-transmitted helminth infections and associated risk factors in three Orang Asli tribes in Peninsular Malaysia. Sci Rep. 2014;4:4101. doi: 10.1038/srep04101 24525479 PMC3924211

[11] Shumbej T, Belay T, Mekonnen Z, Tefera T, Zemene E. Soil-Transmitted Helminths and Associated Factors among Pre-School Children in Butajira Town, South-Central Ethiopia: A Community-Based Cross-Sectional Study. PLOS ONE. 2015;10(8):e0136342. doi: 10.1371/journal.pone.0136342 PMC4548951 26305361

[12] Lwanga F, Kirunda BE, Orach CG. Intestinal helminth infections and nutritional status of children attending primary schools in Wakiso district, central Uganda. Int J Environ Res Public Health. 2012;9(8):2910–21. 23066405 10.3390/ijerph9082910 PMC3447595

[13] Hailegebriel T, Nibret E, Munshea A. Prevalence of Soil-Transmitted Helminth Infection Among School-Aged Children of Ethiopia: A Systematic Review and Meta-Analysis. Infect Dis (Auckl). 2020;13:1178633720962812. doi: 10.1177/1178633720962812 33088182 PMC7543112

[14] Chelkeba L, Mekonnen Z, Emana D, Jimma W, Melaku T. Prevalence of soil-transmitted helminths infections among preschool and school-age children in Ethiopia: a systematic review and meta-analysis. Glob Health Res Policy. 2022;7(1):9. doi: 10.1186/s41256-022-00239-1 35307028 PMC8935818

[15] Geleto GE, Kassa T, Erko B. Epidemiology of soil-transmitted helminthiasis and associated malnutrition among under-fives in conflict affected areas in southern Ethiopia. Trop Med Health. 2022;50(1):44. doi: 10.1186/s41182-022-00436-1 35818081 PMC9275057

[16] Assemie MA. High burden of undernutrition among primary school-aged children and its determinants in Ethiopia: A systematic review and meta-analysis. 2020. 10.1186/s13052-020-00881-w PMC7448995 32847566

[17] Zerdo Z, Yohanes T, Tariku B, Teshome T. Association between nutritional status and soil-transmitted helminthes re-infection among school-age children in Chencha District, Southern Ethiopia: A cross-sectional study. Transl Biomed. 2017;8(2).

[18] World Health Organization, World Health Organization, editors. Preventive chemotherapy to control soil-transmitted helminth infections in at-risk population groups: guideline. Geneva: World Health Organization; 2017. p. 1. 29578660

[19] Asfaw MA, Hailu C, Beyene TJ. Evaluating equity and coverage in mass drug administration for soil-transmitted helminth infections among school-age children in the hard-to-reach setting of southern Ethiopia. Pediatr Health Med Ther. 2021;12:325–33. 10.2147/PHMT.S316194 PMC8275865 34267576

[20] Tesfay K, Yohannes M, Bayisa S. Trend analysis of malaria prevalence in Raya Azebo district, Northern Ethiopia: a retrospective study. BMC Res Notes. 2018;11(1):900. doi: 10.1186/s13104-018-4003-4 30558667 PMC6296012

[21] Projected-population-of-ethiopia-2025. Ethiopian Statistical Service (ESS). Addis Ababa, Ethiopia. https://ess.gov.et/wp-content/uploads/2025/08/projected-population-of-Ethiopia-2025.pdf

[22] Sertse SF, Tamire LC, Liu Y. Modelling climate change risk perception, vulnerability, and adaptation in farming communities: evidence from Raya Azebo district, Ethiopia. Theor Appl Climatol. 2025;156(12):663.

[23] Aemiro A, Menkir S, Girma A. Prevalence of soil-transmitted helminth infections and associated risk factors among school children in Dembecha Town, Ethiopia. Environ Health Insights. 10.1177/11786302241245851 PMC11020722 38628466

[24] Husen EA, Tafesse G, Hajare ST, Chauhan NM, Sharma RJ, Upadhye VJ. Cross-Sectional Study on Assessment of Frequency of Intestinal Helminth Infections and Its Related Risk Factors among School Children from Adola Town, Ethiopia. BioMed Res Int. 2022;2022:1–12. 10.1155/2022/5908938 PMC9015853 35445136

[25] Tariku EZ, Abebe GA, Melketsedik ZA, Gutema BT. Prevalence and factors associated with stunting and thinness among school-age children in Arba Minch Health and Demographic Surveillance Site, Southern Ethiopia. PLoS One. 2018;13(11):e0206659. doi: 10.1371/journal.pone.0206659 30388149 PMC6214544

[26] Demeke G, Fenta A, Dilnessa T. Evaluation of Wet Mount and Concentration Techniques of Stool Examination for Intestinal Parasites Identification at Debre Markos Comprehensive Specialized Hospital, Ethiopia. Infect Drug Resist. 2021;14:1357–62. doi: 10.2147/IDR.S307683 33859481 PMC8043790

[27] Utzinger J, Becker SL, van Lieshout L, van Dam GJ, Knopp S. New diagnostic tools in schistosomiasis. Clin Microbiol Infect. 2015;21(6):529–42. doi: 10.1016/j.cmi.2015.03.014 25843503

[28] Emana D. Prevalence and intensity of soil-transmitted helminths among school-aged children in Sigmo Primary School, Jimma Zone, South-Western Ethiopia. Clin Med Res. 2015;4(4):98.

[29] World Health Organization. Helminth control in school-age children: A guide for managers of control programmes. contre les helminthiases chez les enfants d’âge sc guide à l’intention des programmes de lutte. 2nd ed. 2011;75.

[30] Mekonnen Z, Hassen D, Debalke S, Tiruneh A, Asres Y, Chelkeba L, et al. Soil-transmitted helminth infections and nutritional status of school children in government elementary schools in Jimma Town, Southwestern Ethiopia. SAGE Open Med. 2020;8:2050312120954696. doi: 10.1177/2050312120954696 32953118 PMC7475784

[31] Mengistu B, Maddren R, Collyer B, Anderson RM. Trends in the prevalence and intensity of soil-transmitted helminth (STH) infection in Ethiopia 2000 to 2023: a systematic review. Parasit Vectors. 2025;18(1):340. doi: 10.1186/s13071-025-06928-3 40783768 PMC12335801

[32] Leta GT, Mekete K, Wuletaw Y, Gebretsadik A, Sime H, Mekasha S, et al. National mapping of soil-transmitted helminth and schistosome infections in Ethiopia. Parasit Vectors. 2020;13(1):437. doi: 10.1186/s13071-020-04317-6 32873333 PMC7466696

[33] Seid M, Dejenie T, Tomass Z. Prevalence of intestinal helminths and associated risk factors in rural school-children in Were-Abaye sub district, Tigray region, northern Ethiopia. 2015.

[34] Teklemariam A, Dejenie T, Tomass Z. Infection prevalence of intestinal helminths and associated risk factors among schoolchildren in selected kebeles of Enderta district, Tigray, Northern Ethiopia. J Helminthol. 2023.

[35] Weldesenbet H. Prevalence, infection intensity and associated factors of soil transmitted helminths among primary school children in Gurage zone, South Central Ethiopia: a cross-sectional study design. 2019. 10.1186/s13104-019-4254-8 PMC6469099 30992048

[36] Sumbele IUN, Nkain AJ, Ning TR, Anchang-Kimbi JK, Kimbi HK. Influence of malaria, soil-transmitted helminths and malnutrition on haemoglobin level among school-aged children in Muyuka, Southwest Cameroon: A cross-sectional study on outcomes. PLoS One. 2020;15(3):e0230882. doi: 10.1371/journal.pone.0230882 32226023 PMC7105131

[37] Genet A, Motbainor A, Samuel T, Azage M. Prevalence and associated factors of soil transmitted helminthiasis among school-age children in wetland and non-wetland areas of Blue Nile Basins, northwest Ethiopia: A community-based comparative study. SAGE Open Med. 2021;9:20503121211063354. doi: 10.1177/20503121211063354 34917385 PMC8669120

[38] Molla E, Mamo H. Soil-transmitted helminth infections, anemia and undernutrition among schoolchildren in Yirgacheffee, South Ethiopia. BMC Res Notes. 2018;11(1):585. doi: 10.1186/s13104-018-3679-9 30103797 PMC6090612

[39] Zerdo Z, Bastiaens H, Anthierens S, Massebo F, Masne M, Biresaw G, et al. Prevalence, intensity and endemicity of intestinal schistosomiasis and soil-transmitted helminthiasis and its associated factors among school-aged children in Southern Ethiopia. Sci Rep. 2022;12(1):4586. doi: 10.1038/s41598-022-08333-7 35302056 PMC8931111

[40] Yeshanew S. Soil-transmitted helminthiasis and undernutrition among schoolchildren in Mettu town, Southwest Ethiopia. Sci Rep. 2022. 10.1038/s41598-022-07669-4 PMC8901616 35256678

[41] Samuel F, Demsew A, Alem Y, Hailesilassie Y. Soil transmitted Helminthiasis and associated risk factors among elementary school children in ambo town, western Ethiopia. BMC Public Health. 2017;17(1):791. doi: 10.1186/s12889-017-4809-3 29017470 PMC5634961

[42] Masaku J, Njomo DW, Njoka A, Okoyo C, Mutungi FM, Njenga SM. Soil-transmitted helminths and schistosomiasis among pre-school age children in a rural setting of Busia County, Western Kenya: a cross-sectional study of prevalence, and associated exposures. BMC Public Health. 2020;20(1):356. doi: 10.1186/s12889-020-08485-z 32188444 PMC7079432

[43] Teshale T, Belay S, Tadesse D, Awala A, Teklay G. Prevalence of intestinal helminths and associated factors among school children of Medebay Zana wereda; North Western Tigray, Ethiopia 2017. BMC Res Notes. 2018;11(1):444. doi: 10.1186/s13104-018-3556-6 29973255 PMC6033285

[44] Gitore WA, Ali MM, Yoseph A, Mangesha AE, Debiso AT. Prevalence of soil-transmitted helminthes and its association with water, sanitation, hygiene among schoolchildren and barriers for schools level prevention in technology villages of Hawassa University: Mixed design. PLoS One. 2020;15(9):e0239557. doi: 10.1371/journal.pone.0239557 32970747 PMC7514018

[45] Sturrock SL, Yiannakoulias N, Sanchez AL. The Geography and Scale of Soil-Transmitted Helminth Infections. Curr Trop Med Rep. 2017;4(4):245–55.

[46] Kiiti RW, Omukunda EN, Korir JC. Risk Factors Associated with Helminthic Intestinal Infection in Lurambi Subcounty, Kakamega, Kenya. J Parasitol Res. 2020;2020:1–9. 10.1155/2020/8810519 PMC7790573 33489319

[47] Degarege D, Degarege A, Animut A. Undernutrition and associated risk factors among school age children in Addis Ababa, Ethiopia. BMC Public Health. 2015;15:375. doi: 10.1186/s12889-015-1714-5 25879705 PMC4411785

[48] Asmare B, Taddele M, Berihun S, Wagnew F. Nutritional status and correlation with academic performance among primary school children, northwest Ethiopia. BMC Res Notes. 2018;11(1):805. doi: 10.1186/s13104-018-3909-1 30413190 PMC6230243

[49] Yebyo H g WZ, Gesesew H a MK. Assessment of adolescents’ undernutrition level among school students in eastern Tigray, Ethiopia: A cross-sectional study. J Nutr Food Sci. 2015;5(5).

[50] Berhe K, Gebremariam G. Magnitude and associated factors of undernutrition (underweight and stunting) among school adolescent girls in Hawzen Woreda (District), Tigray regional state, Northern Ethiopia: Cross-sectional study. BMC Res Notes. 2020;13(1):59. doi: 10.1186/s13104-020-4926-4 32029003 PMC7006198

[51] Berhanu A, Garoma S, Arero G, Mosisa G. Stunting and associated factors among school-age children (5-14 years) in Mulo district, Oromia region, Ethiopia. SAGE Open Med. 2022;10:20503121221127880. doi: 10.1177/20503121221127880 36212231 PMC9536101

