# Supplementary material for: Soil-transmitted helminth infections and nutritional status of school-age children, in Mekhoni town, Tigray, Ethiopia
Source: PLoS Negl Trop Dis. 2026 Feb 3;20(2):e0013932. doi: 10.1371/journal.pntd.0013932 (PMC12890091; doi:10.1371/journal.pntd.0013932)
Supplement: S1 File — (DOCX) [file pntd.0013932.s001.docx]

**Annex 3: Questionnaire English and Tigrigna version.**

**Questionnaire English version.**

Mekelle University, College Of Health Sciences, School Of Medicine Biomedical Division, Department Of Parasitology And Entomology.

Name of data collector__________________ Date of data collection ____________

School name____________________

1. **Participant identification**

**Code number----------------**

**Socio-demographic characteristics**:

1. Age in years_______
2. Sex A. Male B. Female
3. Religion A. Orthodox B. Muslim C. Protestant D. Catholic
4. Educational level

A. Grade 1 B. Grade 2 C. Grade 3 D. Grade 4

E. Grade 5 F. Grade 6 G. Grade 7 H. Grade 8

1. Fathers occupation A. Merchant B. Farmer C. Civil servant D. Daily labourer
2. Father educational level A. No formal education B. 1-8 C. ≥9
3. Mother occupation A. Merchant B. civil servant C. housewife D. Daily labourer
4. Mother educational level A. No formal education B. 1-8 C. ≥9
5. Family monthly income A. <1000 B. 1000-2000 C. > 2000
6. Family size A. 1-5 B.5 C. 6 D.>6

**Hygiene practice and environmental factors**

1. Do you wash your Hands before meal? A. Yes B. No
2. If your answer is yes for number 11 when? A. Sometimes B. Always
3. Do you have a Toilet? A. Yes B. No
4. If toilet is available what type of toilet is there? A. Traditional pit latrine B. Improved ventilated C.Water flush
5. Where do you defecate? A. in toilet B. open defecation
6. Do you wash your hands after visiting toilet? A. Yes B. No
7. If yes for number 16 how frequent do you wash? A. always B. sometimes
8. If yes for number 16 how do you wash. A. Using water only B. Using water and soap C. Using ash
9. Finger nail cleanness A. Clean B. Not Clean
10. Are finger nails of participant trimmed? A. Yes B. No
11. How is your shoe wearing habit? A. Sometimes B. Always
12. Is there any Habit of eating unwashed/under cooked vegetables A. Yes B. No
13. What is your Water source for drinking? A. Well water B. River C. Tap water D. Purified water (Bottled)
14. What is your Water source for washing clothes? A. Well water B. River C. Tap water D. Rain water
15. What are your Water sources for bathing? A. Well water B. River C. Tap water D. Rain water
16. How is your solid waste disposal habit A. Burry underground B. Open field C. Incinerate D. By garbage collectors from municipality

**Questionnaire for interview (Tigrigna version) ትግርኛ ቅዳሕ ቃለ መሕትት**

ሽም ሓታታይ____________________ ዕለት__________

ሽም ቤት ትምህርቲ ________________________

**መለለይ መንነት ተሳታፋይ**

መለለይ ቁፅሪ:--------------

1. ዕድመ:-----------------
2. ፆታ: 1.ተባዕታይ 2. ኣንስታይ
3. ሃይማኖት 1. አርቶዶክስ 2. ሙስሊም 3. ፕሮቲስታንት 4. ካቶሊክ
4. ደረጃ ትምህርቲ

1. 1ይ 2. 2ይ 3. 3ይ 4. 4ይ

5. 5ይ 6. 6ይ 7. 7ይ 8. 8ይ

1. ኩነታት ስራሕ ኣቦ 1.ነጋዳይ 2. ሓረስታይ 3.መንግስቲ ሰራሕተኛ 4. መዓልታዊ ሰራሕተኛ
2. ደረጃ ትምህርት ኣቦ 1. ስሩዕ ት/ቲ ዘይብሉ 2. 1-8. 3. ≥ 9
3. ኩነታት ስራሕ ኣዶ. 1. ነጋዲት. 2. ሰራሕተኛ መንግስት. 3. ናይ ገዛ ስራሕ. 4.መዓልታዊ ሰራሕተኛ
4. ደረጃ ት/ቲ ኣዶ. 1. ስሩዕ ት/ቲ ዘይብላ. 2. 1-8. 3. ≥9
5. ወርሓዊ እቶት/ብብር/ 1. ትሕቲ 1000 2. ካብ 1000-2000 3.ልዕሊ 2000
6. በዝሒ ስድራ 1. 1-5 2.5 3.6 4. ልዕሊ 6
7. ቅድሚ ምግቢ ምምጋብካ/ኪ ኢድካ/ኪ ትሕፀብ/ቢ ዶ? 1. እወ 2.አይፋሉን
8. መልሲ ቁፅሪ 11 እወ እንተኾይኑ መዓዝ ትሕፀብ ? 1. ሓደ ሓደ ግዜ 2. ኩሉ ግዜ ይሕፀብ
9. ሽንትቤት (ሽቓቕ) ኣለኩም ዶ? 1.እወ 2. ኣይፋሉን
10. ቁፅሪ 13 እወ እንተኮይኑ እንታይ ዓይነት ሽንትቤት (ሽቓቕ) ? 1. ናይ ጉድጔድ ሽንቲ ቤት መውፅኢ ኣየር ዘይብሉ

2. ናይ ጉድጔድ ሽንቲ መውፅኢ ኣየር ዘለዎ. 3.ተሓፃብን መውፅኢ አየር ዘለዎን

1. ኣብ ምንታይ ኢካ ትፍትን? 1. ኣብ ሽንትቤት 2. ኣብ ደገ
2. ድሕሪ ሽቓቕ ኢድካ/ኪ ትሕፀብ/ቢ? 1. እወ 2. ኣይፋሉን
3. ቁፅሪ 16 እወ እንተኾይኑ መዓዝ መዓዝ ትሕፀብ/ቢ? 1 .ኩሉግዜ 2. ሓደ ሓደ ግዜ
4. ቁፅሪ 16 እወ ተኾይኑ ብምንታይ/ብከመይ ትሕፀብ/ቢ

1. ብማይ 2.ብሳሙናን 3. ብሓሙክሽቲ

1. ፅሬት ፅፍሪ /ብትዕዝብቲ/ ፅሩይ ድዩ? 1. እወ 2.ኣይፋሉን
2. ፅፍሪ ኢድ ተፀፊሩ/ተስተካኪሉ/ ዶ /ብትዕዝብቲ/ 1. እወ 2.ኣይፋሉን
3. ጫማ እግሪ ኣብ ምግባር ከመይ ኢካ/ኪ? 1.ሓደ ሓደ ግዜ እየ ዝገብር 2.ኩሉ ግዜ ይገብር
4. ዘይበሰለ ወይ ዘይተሓፀበ ኣትክልቲ በሊዕካ ዶ ትፈልጥ? 1.እወ 2.ኣይፋሉን
5. ፍልፍል ንመስተ ትጥቀምሉ ማይኩም እንታይ እዩ 1. ጉድጓድ ማይ 2. ሩባ 3.ማይቡንባ 4.ዕሹግ ዝተፃረየ ማይ
6. ንመሕፀቢ ክዳን ማይ ካብ ምንታይ ትጥቀሙ 1. ጉድጋድ ማይ 2. ሩባ 3. ማይ ቡንባ 4. ማይ ዚንጎ
7. ፍልፍል መሕፀቢ ሰዉነት ማይ ካብ ምንታይ ትረክቡ 1. ጉድጓድ ማይ 2. ሩባ 3. ማይ ቡንባ 4. ማይ ዚንጎ
8. ጉሓፍ ከመይ ትጉሕፉ

1. ትሕቲ መሬት ብምቕባር 2. ኣብ ክፍቲ ቦታ

3. ኣብ መቃፀሊ ጉሓፍ ብምቅፃል 4. ኣኪብካ ናብ ጉሓፍ ዝእክባ መኪና ምሃብ
