# Supplementary material for: Soil-transmitted helminth infections and nutritional status of school-age children, in Mekhoni town, Tigray, Ethiopia
Source: PLoS Negl Trop Dis. 2026 Feb 3;20(2):e0013932. doi: 10.1371/journal.pntd.0013932 (PMC12890091; doi:10.1371/journal.pntd.0013932)
Supplement: S1 Table — (DOCX) [file pntd.0013932.s002.docx]

Table 1: Socio-demographic, hygienic, and environmental characteristics of school-age children, Mekhoni town, Tigray, Ethiopia May 2024 to March 2025 (N=277).

| Variables | Category | Frequency (%) | | STH positive | STH negative | | | COR | p-value |
| --- | --- | --- | --- | --- | --- | --- | --- | --- | --- |
| School | Degol | 176(63.5) | | 30 | 146 | | | 1.17 | 0.63 |
|  | Hayelom Araya | 101(36.5) | | 15 | 86 | | | 1 |  |
| Sex | Male | 166(59.9) | | 135 | 31 | | | 1.59 | 0.18 |
|  | Female | 111(40.1) | | 97 | 14 | | | 1 |  |
| Age in years | 5-10 | 120(43.3) | | 22 | 98 | | | 1.3 | 0.4 |
|  | 11-14 | 157(56.7) | | 23 | 134 | | | 1 |  |
| Religion | Orthodox | 234(84.5) | | 38 | 196 | | | 0.99 | 0.99 |
|  | Muslim | 43(15.5) | | 7 | 36 | | | 1 |  |
| Grade | 1-4 | 150(54.2) | | 28 | 122 | | | 1.49 | 0.24 |
|  | 5-8 | 127(45.8) | | 17 | 110 | | | 1 |  |
| Monthly family income (ETB) | <1000 | 39(14.1) | | 7 | 32 | | | 1.13 | 0.81 |
|  | 1000-2000 | 25(9) | | 3 | 22 | | | 0.69 | 0.57 |
|  | >2000 | 213(76.9) | | 35 | 178 | | | 1 |  |
| Family size | ≤5 | 137(49.5) | | 14 | 123 | | | 0.4 | 0.008 |
|  | >5 | 140(50.5) | | 31 | 109 | | | 1 |  |
| Mother’s educational status | No formal education | 97(35) | | 16 | 81 | | | 1.17 | 0.7 |
|  | 1-8 | 97(35) | | 17 | 80 | | | 1.26 | 0.57 |
|  | ≥9 | 83(30) | | 12 | 71 | | | 1 |  |
| Mother’s occupation | Merchant | 124(44.8) | | 22 | 102 | | | 0.92 | 0.95 |
|  | Civil servant | 65(23.5) | | 9 | 56 | | | 0.57 | 0.7 |
|  | Housewife | 61(22) | | 9 | 52 | | | 0.65 | 0.76 |
|  | Daily labourer | 27(9.7) | | 5 | 22 | | | 1 |  |
| Father’s educational status | No formal education | 103(37.2) | | 17 | 86 | | | 0.98 | 0.99 |
|  | 1-8 | 90(32.5) | | 14 | 76 | | | 0.84 | 0.92 |
|  | ≥9 | 84(30.3) | 14 | | | | 70 | 1 |  |
| Fathers’ occupation | Merchant | 102(36.8) | 12 | | | | 90 | 0.52 | 0.16 |
|  | Farmer | 58(20.9) | 10 | | | | 48 | 0.81 | 0.68 |
|  | Civil servant | 68(24.5) | 13 | | | | 55 | 0.92 | 0.86 |
|  | Daily labourer | 49(17.7) | 10 | | | | 39 | 1 |  |
| Wash hands before meal | Yes | 259(93.5) | 41 | | | | 218 | 0.66 | 0.48 |
|  | No | 18(6.5) | 4 | | | | 14 | 1 |  |
| Frequency of washing hand before meal | Sometimes | 31(11.2) | 5 | | | | 26 | 1.02 | 0.96 |
|  | Always | 228(82.3) | 36 | | | | 192 | 1 |  |
| Wash hands after toilet | Yes | 271(97.8) | 44 | | | | 227 | 0.97 | 0.98 |
|  | No | 6(2.2) | 1 | | | | 5 | 1 |  |
| Frequency of washing hands after toilet | Always | 217(78.3) | 34 | | | | 183 | 0.6 | 0.8 |
|  | Sometimes | 54(19.5) | 10 | | | | 44 | 1 |  |
| Using water and/or soap after toilet | Using water only | 92(33.2) | 27 | | | | 65 | 3.96 | 0.001 |
|  | Using water and soap | 179(64.6) | 17 | | | | 162 | 1 |  |
| Fingernail cleanness | Clean | 202(72.9) | 21 | | | | 181 | 0.25 | 0.001 |
|  | Not clean | 75(27.1) | 24 | | | | 51 | 1 |  |
| Trimming fingernails | Trimmed | 175(63.2) | 20 | | | | 155 | 0.397 | 0.005 |
|  | Not trimmed | 102(36.8) | 25 | | | | 77 | 1 |  |
| Shoe wearing habit | Sometimes | 2(0.7) | 1 | | | | 1 | 5.3 | 0.24 |
|  | Always | 275(99.3) | 44 | | | | 231 | 1 |  |
| Habit of eating unwashed / undercooked vegetables | Yes | 31(11.2) | 6 | | | | 25 | 1.27 | 0.6 |
|  | No | 246(88.8) | 39 | | | | 207 | 1 |  |
| Availability of toilet | Yes | 256(92.4) | 42 | | | | 214 | 1.2 | 0.8 |
|  | No | 21(7.6) | 3 | | | | 18 | 1 |  |
| Type of toilet | Traditional pit latrine | 138(49.8) | 24 | | | | 114 | 1.17 | 0.65 |
|  | Ventilated improved | 118(42.6) | 18 | | | | 100 | 1 |  |
| Solid waste disposal habit | Burry underground | 17(6.1) | 4 | | | 13 | | 1.9 | 0.28 |
|  | Open field | 19(6.9) | 5 | | | 14 | | 2.2 | 0.15 |
|  | Incinerate | 17(6.1) | 5 | | | 12 | | 2.6 | 0.09 |
|  | By municipality | 224(80.9) | 31 | | | 193 | | 1 |  |
| Water source for drinking | Well water | 77(27.8) | 14 | | | 63 | | 1.2 | 0.59 |
|  | Tap water | 200(72.2) | 31 | | | 169 | | 1 |  |
| Water source for bathing | Well water | 77(27.8) | 14 | | | 63 | | 1.2 | 0.59 |
|  | Tap water | 200(72.2) | 31 | | | 169 | | 1 |  |
| Water source for washing cloth | Well water | 77(27.8) | 14 | | | 63 | | 1.2 | 0.59 |
|  | Tap water | 200(72.2) | 31 | | | 169 | | 1 |  |
